# Performance of fluoro-2-deoxy-D-glucose positron emission tomography-computed tomography imaging for lymph node staging in bladder and upper tract urothelial carcinoma: a systematic review

**DOI:** 10.1080/2090598X.2020.1858012

**Published:** 2020-12-10

**Authors:** Abdulmajeed Aydh, Mohammad Abufaraj, Keiichiro Mori, Fahad Quhal, Benjamin Pradere, Reza Sari Motlagh, Hadi Mostafaei, Pierre I. Karakiewicz, Shahrokh F. Shariat

**Affiliations:** a Department of Urology, Comprehensive Cancer Center, Medical University of Vienna, Vienna, Austria; b Department of Urology, King Faisal Medical City, Abha, Saudi Arabia; c Division of Urology, Department of Special Surgery, Jordan University Hospital, the University of Jordan, Amman, Jordan; d Department of Urology, Jikei University School of Medicine, Tokyo, Japan; e Department of Urology, King Fahad Specialist Hospital, Dammam, Saudi Arabia; f Department of Urology, University Hospital of Tours, To Urs, France; g Men’s Health and Reproductive Health Research Center, Shahid Beheshti University of Medical Sciences, Tehran, Iran; h Research Center for Evidence Based Medicine, Tabriz University of Medical Sciences, Tabriz, Iran; i Cancer Prognostics and Health Outcomes Unit, Division of Urology, University of Montreal Health Center, Montreal, Canada; j Departments of Urology, Weill Cornell Medical College, New York, NY, USA; k Department of Urology, University of Texas Southwestern, Dallas, TX, USA; l Department of Urology, Second Faculty of Medicine, Charles University, Prague, Czech Republic; m European Association of Urology Research Foundation, Arnhem, The Netherlands; n Department of Urology, Karl Landsteiner Institute, Vienna, Austria; o Institute for Urology and Reproductive Health, Sechenov University, Moscow, Russia

**Keywords:** Bladder, cancer, diagnostic, accuracy, imaging, urothelial

## Abstract

**Objective**: To evaluate the current literature on the accuracy of fluoro-2-deoxy-*D*-glucose positron emission tomography-computed tomography (FDG PET-CT) for lymph node (LN) staging in urothelial carcinoma (UC), as robust evidence on the value of this technology in UC is still lacking.

**Methods**: The Medical Literature Analysis and Retrieval System Online (MEDLINE)/PubMed, Cochrane Library, and Scopus databases were searched for eligible studies. We included all original studies evaluating FDG PET-CT in bladder or upper tract UC. The search results were restricted to the English language, and included prospective and retrospective studies without time restriction. We included only studies reporting the sensitivity and specificity of FDG PET-CT in detecting UC LN metastases.

**Results**: We identified 23 articles meeting our inclusion criteria. In the preoperative setting, the sensitivity of FDG PET-CT for detecting LN metastases in patients with bladder cancer was widely variable ranging from 23% to 89%; the specificity ranged from 81% to 100%; and the overall accuracy ranged from 65% to 89%. During bladder cancer monitoring the sensitivity for detecting LN metastases ranged from 75% to 92% and the specificity ranged from 60% to 92%. The sensitivity for LN staging in upper tract UC ranged between 82% and 95%, with a specificity of 84–91%.

**Conclusion**: Despite the inconsistencies in sensitivity between the reports, FDG PET-CT seems to have a high specificity for LN staging in patients with UC. Future prospective, well-designed studies are necessary to evaluate the role of FDG PET-CT in UC management.

**Abbreviations:** FDG: fluoro-2-deoxy-*D*-glucose; LN: lymph node; PET: positron emission tomography; PRISMA: Preferred Reporting Items for Systematic Reviews and Meta-analyses; PSMA: prostate-specific membrane antigen; (N)(P)PV: (negative) (positive) predictive value; QUADAS-2: Quality Assessment of Diagnostic Accuracy Studies-2; SUV_max_: maximum standard uptake value; (UT)UC: (upper urinary tract) urothelial carcinoma

## Introduction

Urothelial carcinoma (UC) arises from the urothelium of the urinary tract potentially affecting the urinary bladder in 90–95% of cases and, less commonly, the upper urinary tract in 5–10% (i.e. ureters and pyelocalyceal system) [1–3]. In the United States, bladder UC alone accounts for 81 400 cases per year, with 17 980 deaths, while ureteric UC accounted for 3970 cases and 1010 deaths per year [4]. While diagnosis with conventional techniques such as cytology, endoscopy, biopsy, and histopathological evaluation of these tumours is established, accurate UC staging is still challenging [5–8]. Various imaging techniques have been proposed for accurate staging of UC in order to improve patient counselling and treatment planning [9,10]. Conventional imaging techniques, e.g. CT urography and magnetic resonance urography, have poor performance to impact clinical decision-making regarding lymph node (LN) status [1,2]. The overall accuracy of the CT scan was estimated to be between 56% and 90%, with an under- and over-staging rate of 39% and 6%, respectively [11,12]. For MRI, the reported sensitivity for the staging of UC of the bladder in terms of extravesical extension was 60% [13]. Such performance is considered inadequate for proper treatment planning, given the sensitive and varying treatment algorithms.

In the last decade, positron emission tomography (PET) with the glucose analogue fluoro-2-deoxy-*D*-glucose (FDG) has been used to improve the staging and monitoring of patients with different malignancies, e.g. lung, breast, colorectal, as well as UC [14–16]. PET measures the metabolic activity of the target cells, but suffers from an inaccuracy to capture anatomical details. Thus, a combination of PET and other imaging techniques, e.g. CT, can overcome the limitations of both technologies [17]. Metastatic burden plays an essential role in choosing the appropriate treatment, especially in the era of metastases-targeted therapies and salvage surgery [18]. FDG PET-CT has, indeed, been recently recommended as a non-invasive method for the diagnosis and staging of UCs [19]. However, robust evidence on the accuracy of this technology is still lacking. In the present review, we aimed to summarise the current evidence regarding the accuracy of FDG PET-CT for LN staging and monitoring of bladder and upper tract (UT) UC.

## Methods

This systematic review aimed to evaluate the diagnostic accuracy of the FDG PET-CT technique in the staging and monitoring of UC. We performed this review according to the Preferred Reporting Items for Systematic Reviews and Meta-analyses (PRISMA) statement [20]. A comprehensive systematic literature search was independently performed by two authors. The search results were restricted to the English language and without time restriction. We excluded case series, case reports, and studies reporting on <10 patients. The participants in the included studies were adult patients with UC of the bladder or UTUC who were clinically followed to obtain a diagnosis, which was considered as the confirmation for FDG PET-CT accuracy in LN detection. Moreover, we included studies that reported the sensitivity and specificity of FDG PET-CT in detecting UC metastases. The outcomes of interest were diagnostic performance, particularly sensitivity, specificity, positive (PPV) and negative predictive value (NPV), and overall accuracy.

### Data sources

Electronic databases were searched, including the Medical Literature Analysis and Retrieval System Online (MEDLINE)/PubMed, and Scopus. The databases of systematic reviews, e.g. the Cochrane libraries and Center for Reviews and Dissemination, were screened for eligible primary studies in April 2020, and saved for further screening and in-depth reading. The following keywords were used: ‘transitional cell carcinoma’, ‘urothelial cancer’, ‘urologic neoplasms’, ‘upper tract’, ‘upper tract urothelial cancer’, ‘bladder cancer’, OR ‘urinary tract cancer’, ‘urothelial cancer’, ‘FDG’ OR ‘Fluorodeoxyglucose’, ‘FDG-PET’, ‘PET-CT’.

**Figure 1.**
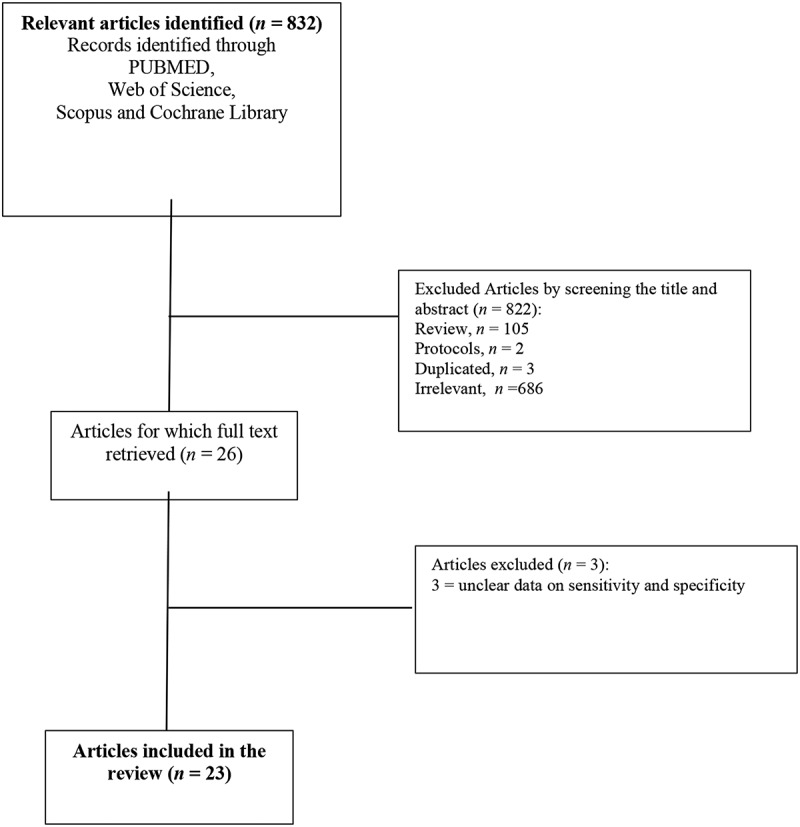
Flow diagram of the included studies in the systematic review

The flow of the information through the different stages of a systematic review (primary screening, secondary screening, and inclusion stage) is shown in the PRISMA flow chart (Figure 1).

### Data collection and analysis

Data from each study were extracted by two independent reviewers. Data were collected from the included studies into data extraction tables, which were designed for this review and contained: author name, year of publication, study design, sample size, characteristics of the imaging technique, performance measures (sensitivity, specificity, PPV and NPV, overall accuracy), and any relevant comment of the included study.

### Risk of bias assessment

The risk of bias was evaluated according to the Quality Assessment of Diagnostic Accuracy Studies-2 (QUADAS-2) tool. This tool is based on four domains: patient selection, index test, reference standard, and the timing of reference test. Also, the QUADAS-2 tool assessed applicability concerns of (patient selection, index test, and reference standard) (Table 1 [10,21–41]).

**Table 1.** Quality appraisal for included studies using the QUADAS-2 tool. Green = low, i.e. risk of bias low or concerns about applicability low. Red = high or uncertain, i.e. risk of bias high/uncertain or concerns about applicability high/uncertain

Reference	Risk of bias				Applicability concerns		
	Patient selection	Index test	Reference standard	Flow and timing	Patient selection	Index test	Reference standard
Dason et al., [34]	+	+	+	–	+	+	+
Girard et al., [28]	+	–	–	–	+	+	+
Vind-Kezunovic et al., [35]	+	+	–	–	+	+	+
Pichler et al., [33]	–	–	+	+	+	+	+
Soubra et al., [39]	+	+	–	–	+	+	+
Jeong et al., [40]	+	+	–	–	+	+	+
Aljabery et al., [32]	+	+	–	–	+	+	+
Goodfellow et al., [31]	+	+	–	+	+	+	+
Rouanne et al., [26]	+	–	–	+	+	+	+
Hitier‐Berthault et al., [27]	+	+	+	–	+	+	+
Jensen et al., [41]*	+	–	–	–	+	+	+
Apolo et al., [24]*	+	+	–	–	+	+	+
Lodde et al., [25]	–	–	–	–	+	+	+
Swinnen et al., [30]	+	–	–	+	+	+	+
Kibel et al., [23]	–	+	+	–	–	+	+
Drieskens et al., [29]	+	+	+	+	–	+	+
Öztürk et al., [38]	+	+	–	–	+	+	+
Yang et al., [37]*	+	+	–	–	+	+	+
Jadvar et al., [36]	+	–	–	+	+	+	+
Tanaka et al., [21]	–	+	+	–	–	+	+
Asai et al., [22]	+	+	+	+	–	+	+
Voskuilen et al., [10]	+	+	–	–	+	+	+

## Results

The initial search resulted in 822 articles, from which 23 articles met our inclusion criteria: 20 articles evaluated UC of the bladder, whereas three addressed UTUC [10,21,22]. Regarding the main outcome of the included studies, different measures were used to estimate the diagnostic value of the FDG PET-CT technique in the diagnosis or staging of UC. Sensitivity and specificity were reported in all included studies. The summary of the risk of bias and applicability concerns; overall, the quality of the studies was deemed satisfactory.

### Role of FDG PET-CT in UC of the bladder

Between 2009 and 2014, several studies reported favourable results of FDG PET-CT for LN detection in patients with bladder UC. Kibel *et al*. [23] and Apolo *et al*. [24] evaluated LN detection of FDG PET-CT and showed high sensitivity of (70% and 92%, respectively) and specificity of (94% and 81%, respectively). Likewise, higher sensitivity for FDG PET-CT (57%) as compared with CT alone (33%) for detecting LN metastases was reported by Lodde *et al*. [25]. Rouanne *et al*. [26] evaluated 102 patients prospectively and revealed moderate sensitivity and high specificity for detecting LN using FDG PET-CT (50% and 97%, respectively). A similar conclusion was reached by Hitier‐Berthault *et al*. [27], who prospectively compared FDG PET-CT and CT alone in 52 patients, and reported a higher sensitivity of FDG PET-CT for LN detection (36% vs 9%). A similar pattern of favourable results was obtained by Girard *et al*. [28] in that FDG PET-CT had significant diagnostic accuracy compared to CT alone, which was 84%, as well as sensitivity and specificity that were 29% and 97%, respectively.

However, several studies have reported less promising results. Drieskens *et al*. [29] found a comparable sensitivity for FDG PET-CT and CT alone in detecting LN metastasis (50% and 42%, respectively). Swinnen *et al*. [30] observed similar results, a sensitivity of 46% for both FDG PET-CT and CT alone, as well as no considerable benefit of specificity for detecting LN metastasis (97% and 92%, respectively). In addition, a large study that included 233 patients [31], showed 69% sensitivity of FDG PET-CT as compared with 41% for CT alone, which was not significant enough to justify the additional cost according to the authors’ opinion. Aljabery *et al*. [32], also showed no advantage of PET-CT in terms of sensitivity and specificity (41% and 86%, respectively) as compared to CT alone (41% and 89%, respectively). Along with the previous study, Pichler *et al*. [33] found comparable sensitivity and specificity results for FDG PET-CT (64% and 86%, respectively) and CT alone (46% and 92%, respectively). Moreover, in 2020, Dason *et al*. [34] retrospectively evaluated 208 patients and found very poor sensitivity of FDG PET-CT for detecting LN metastases, which was between 7% and 23%, even though they found high specificity from 89% to 99%.

Vind-Kezunovic *et al*. [35] evaluated the maximum standard uptake value (SUV_max_) on FDG-PET scans in 131 patients with bladder cancer. The SUV_max_ >2 FDG-PET scans had a sensitivity and specificity of 79% and 66%, respectively. In contrast, a SUVmax of >4 FDG-PET had a sensitivity and specificity of 61% and 84%, respectively, which may help in identifying patients with a poor prognosis (Table 2 [23–35,39-41]).

**Table 2.** ^18^F-FDG PET-CT in LN staging in patients with UC

Reference	No. of patients	Study type	Accuracy (per patient), %	Sensitivity (per patient), %	Specificity (per patient), %	PPV, %	NPV, %	Comments
Dason et al., [34]	208	Retro.	–	7–23	89–99	25–37	75–83	
Girard et al., [28]	61	Retro.	82–84	CT 25, PET 29, PET-CT 29	CT 91, PET 94, PET-CT 97	70	85	PPV increase with PET-CT 8/10 (80%) and NPV same
Vind-Kezunovic et al., [35]	131	Prosp.	–	79.4 (SUV_max_ >2); 61.8 (SUV_max_ >4)	66.5 (SUV_max_ >2); 84.5 (SUV_max_ >4)	–	–	A higher SUV_max_ (e.g. SUV_max_ >4) can be of clinical importance aiding in differentiation between patients with a poor prognosis
Pichler et al., [33]	70	Retro.	CT 84, PET 85, PET-CT 83	CT 46, PET 55, PET-CT 64	CT 92, PET 90, PET-CT 86	46.7	92.7	–
Soubra et al., [39]	78	Retro.	PET-CT 89.7	PET-CT 56.3	PET-CT 98.4	90	89.7	No comparison with CT
Jeong et al., [40]	61	Prosp.	–	PET-CT 47.1, CT 29.4	PET-CT 93.2 CT 97.7	72.7	82.0	–
Aljabery et al., [32]	54	Prosp.	–	PET-CT 41, CT 41	PET-CT 86 CT 89	58.3	76.2	–
Goodfellow et al., [31]	233	Retro.	PET 82, CT 83, PET-CT 87	PET 46, CT 46, PET-CT 69	PET 97, CT 98, PET/CT 95	86.4	87.3	PET scan may be useful in selected patients with enlarged pelvic LNs and a small primary bladder tumour, suspected metastases in LNs outside of the pelvic lymphadenectomy window and patients with indeterminate metastases
Rouanne et al., [26]	102	Prosp.	PET-CT 85.3	PET-CT 50	PET-CT 97.4	86.7	85.1	Improved diagnostic efficacy of PET-CT for LN staging in patients staged N0 with conventional cross-sectional imaging
Hitier‐Berthault et al., [27]	52	Prosp.	CT 55.7, PET-CT 65.4	CT 9.1, PET-CT 36.4	CT 90, PET/CT 86.7	66.7	65	–
Jensen et al., [41]*	18	Retro.	–	MRI 0, PET-CT 33	MRI 80, PET-CT 93.3	50	87.5	–
Apolo et al., [24]*	57	Prosp.	–	PET-CT 92	PET-CT 81	–	–	FDG PET-CT compared with conventional MRI or CT alone, detected more lesions detected in 40% of patients
Lodde et al., [25]	44	Prosp.	–	CT 33, PET-CT 57	CT 100, PET-CT 100	100	66.7	–
Swinnen et al., [30]	51	Prosp.	CT 80, PET-CT 84	CT 46, PET-CT 46	CT 92, PET-CT 97	85.7	84.1	–
Kibel et al., [23]	43	Prosp.	–	PET-CT 70	PET-CT 94	77.8	90.9	FDG PET-CT detected the occult metastatic disease in seven of 42 patients with negative conventional preoperative evaluations. PET findings were strongly correlated with survival
Drieskens et al., [29]	55	Prosp.	PET 65, PET-CT 78	PET 53, PET-CT 60	PET 72, PET-CT 88	–	–	Sensitivity of FDG PET for LN staging found in this study was comparatively low (50% for PET-CT vs 42% for CT)

Prosp.: prospective; Retro.: retrospective; *MRI, magnetic resonance imaging was included.

### Role of FDG-PET in monitoring

Jadvar *et al*. [36] retrospectively studied 35 patients and found that the sensitivity of PET-CT was double that of CT alone in detecting metastatic LN and changed the management of 17% of patients. Overall, these findings are consistent with the Apolo *et al*. [24] study that concluded that PET-CT affected the management in 68% of patients, with a sensitivity of 75% and specificity of 92%. Yang *et al*. [37] also reported sensitivity and specificity of PET-CT in patients with recurrent bladder UC of 87% and 89%, respectively. Likewise, Öztürk *et al*. [38] reported a sensitivity of 92% and specificity of 83% using PET-CT in the detection of recurrence after radical cystectomy (Table 3 [24,36–38]).

**Table 3.** ^18^F-FDG PET-CT in re-staging of LN in UC

Reference	No. of patients	Study type	Accuracy (per patient), %	Sensitivity (per patient), %	Specificity (per patient), %	PPV, %	NPV, %	Comments
Öztürk et al., [38]	51	Retro.	90	92	83	94	77	–
Yang et al., [37]*	60	Retro.	–	87.1	89.7	–	–	PET-CT outperformed CT, ultrasound, and MRI in changing management and correctly re-staging UC after surgery
Apolo et al., [24]	25	Prosp.	–	75	92	–	–	PET-CT change management decisions in 68% of patients undergoing PET scans for re-staging
Jadvar et al., [36]	35	Retro.	–	–	–	–	–	PET-CT affected the clinical management in six patients

Prosp.: prospective; Retro.: retrospective;*MRI was included.

### *Role of FDG PET-CT in* UTUC

The performance of FDG PET-CT in the evaluation of UTUC has been examined in three retrospective studies [10,21,22]. Tanaka *et al*. [21] compared the diagnostic accuracy of FDG PET-CT and CT alone in 53 patients for detecting UTUC metastases in primary and recurrent diseases. The authors found that the sensitivity and specificity of FDG PET-CT were comparable to CT alone (95% and 91% respectively for FDG PET-CT, and 82% and 85% respectively for CT). Asai *et al*. [22] investigated the performance of FDG PET-CT in the detection of LNs in a subgroup of 28 patients with UTUC and reported a sensitivity of 60%, with no data on specificity or other measures. Finally, in 2019, Voskuilen *et al*. [10] evaluated the role of FDG PET-CT for the detection of LN metastases in 117 patients with UTUC. Overall, the authors reported a sensitivity and specificity of FDG PET-CT of 82% and 84%, respectively (Table 4 [10,21,22]).

**Table 4.** ^18^F-FDG PET-CT for patients with upper tract UC

Reference	No. of patients	Study type	Accuracy (per patient), %	Sensitivity (per patient), %	Specificity (per patient), %	PPV, %	NPV, %	Comments
Tanaka et al., [21]	53	Retro.	–	95	91	–	–	–
Asai et al., [22]	48	Retro.	–	60	–	–	–	–
Voskuilen et al., [10]	117	Retro.	83.9	82	84	66	92	The presence of suspicious LNs on FDG-PET-CT is associated with worse RFS. But no difference in OS.

Retro.: retrospective.

## Discussion

The introduction of any technique to clinical practice requires proving the diagnostic accuracy with a low risk of harm. Non-invasive diagnostic techniques such as CT or MRI were found to be inadequate in the diagnosis and staging of UC [11]. New techniques with high diagnostic performance will improve the detection of tumours, as well as metastatic deposits guiding surgical intervention and improving survival.

Regarding the LN staging of UC, the variability in the sensitivity measures was more evident than in the monitoring phase. The highest sensitivity was estimated at 95%, while the lowest sensitivity was 7–23% [34]. On the other hand, specificity was generally higher than sensitivity with more consistent values among the included studies, which ranged from 81% [24] to 100% [25]. The PPV, which is more important from a clinical perspective, ranged from 25% to 37% [34], to 100% [25]. Likewise, the NPV ranged from 65% [27] to 92% [33]. Regarding the monitoring of LN in UC, the sensitivity measures among the included studies were higher than those in the staging phase. It ranged from 75% [24] to 90% [38]. The specificity for monitoring of LN in UC was generally lower than that reported in the staging phase. It ranged from 83% [38] to 92% [24]. Generally, the sample size in the monitoring studies was small, except in a study conducted by Lu *et al*. [42], where 236 patients were recruited.

Our present result is consistent with what has been found with the use of PET-CT with radiolabelled prostate-specific membrane antigen (PSMA) for diagnostic assessment of patients with prostate cancer [43]. PSMA PET/CT has revealed a higher sensitivity than traditional methods in detecting metastases in primary staging to detect metastases [44]. A meta-analysis included 298 patients with prostate cancer who underwent traditional imaging studies and PSMA PET-CT, the detection of metastatic LNs was higher by PSMA PET-CT than with CT and MRI (71% sensitivity and 95% specificity) [45].

The ability to exclude false positives in the staging is an advantage of this technique. While in staging tumours, specificity is more important than sensitivity because false positives can be subjected to unnecessary invasive investigations such as biopsies. Nevertheless, missing cancer cases due to low sensitivity may result in delayed treatment and associated adverse effects. Thus, several sequential diagnostic modalities are recommended to improve the net sensitivity of staging LN of UC. These findings demonstrate that studies assessing the role of PET-CT in LN monitoring revealed comparatively high sensitivity and specificity values of PET-CT in surveillance and proved its capability to impact individual treatment plans and change the clinical decision.

Generally, FDG PET-CT has higher diagnostic values in cancer monitoring than in cancer staging [46]. This can be attributed to the anatomical locations and surrounding tissues, which makes it challenging to diagnose LN involvement using imaging techniques. A possible explanation for the low accuracy is the high flow of fluids in the urinary system that interferes with image interpretation. The importance of the scanning protocol was highlighted by Mertens *et al*. [47], who found a sensitivity for cancer detection of 38% in the empty bladder protocol, and 63% in the filled bladder protocol. Adjunct to FDG PET imaging, which detects highly metabolic metastatic LN to the anatomical assessment provided by CT, increases the sensitivity for detecting LNs that are not large enough to be recognised by CT alone. Nevertheless, FDG PET-CT sensitivity is, indeed, adversely affected by minor metastatic lesions that cannot reach the metabolic activity threshold [48]. Furthermore, the low specificity of FDG PET is mainly related to a high rate of false-positive LNs, where the uptake of ^18^F-FDG is caused by inflammatory states and not by cancer.

The approach utilised suffers from the limitation that the included studies are mostly retrospective. Other limitations include heterogeneous patient populations, non-standardisation of techniques, variable follow-up durations, and different confirmatory tests. Furthermore, most of the included studies lacked statistical power due to small samples, which are far lower than the sample size recommended for diagnostic studies. There is a need for further well-designed studies to accurately determine the diagnostic accuracy of FDG PET-CT in patients with UC.

## Conclusions

The use of FDG PET-CT appears to have a high sensitivity for both UTUC and bladder UC in the detection of LN metastasis. Despite the high specificity of FDG PET-CT, the detection of LNs is still in the range of conventional images. Albeit the role of PET-CT could be considered as promising, more research is required to establish it as the imaging modality of choice, or at least to recommend its routine use in a selected group of patients.
